# Usefulness of second ^131^I treatment in biochemical persistent differentiated thyroid cancer patients

**DOI:** 10.1530/ETJ-23-0052

**Published:** 2023-10-18

**Authors:** Carla Gambale, Alessandro Prete, Lea Contartese, Liborio Torregrossa, Francesca Bianchi, Eleonora Molinaro, Gabriele Materazzi, Rossella Elisei, Antonio Matrone

**Affiliations:** 1 Department of Clinical and Experimental Medicine, Unit of Endocrinology, University Hospital of Pisa, Pisa, Italy; 2 Department of Surgical, Medical, Molecular Pathology and Critical Area, Anatomic Pathology Section, University Hospital of Pisa, Pisa, Italy; 3 Department of Nuclear Medicine, University Hospital of Pisa, Pisa, Italy; 4 Department of Surgical, Medical, Molecular Pathology and Critical Area, Unit of Endocrine Surgery, University Hospital of Pisa, Pisa, Italy

**Keywords:** differentiated thyroid cancer, biochemical incomplete response, indeterminate response, radioiodine, thyroglobulin, thyroglobulin antibodies

## Abstract

**Background:**

Second ^131^I treatment is commonly performed in clinical practice in patients with differentiated thyroid cancer and biochemical incomplete or indeterminate response (BiR/InR) after initial treatment.

**Objective:**

The objective of the is study is to evaluate the clinical impact of the second ^131^I treatment in BiR/InR patients and analyze the predictive factors for structural incomplete response (SiR).

**Patients and methods:**

One hundred fifty-three BiR/InR patients after initial treatment who received a second ^131^I treatment were included in the study. The clinical response in a short- and medium- long-term follow-up was evaluated.

**Results:**

After the second ^131^I treatment (median 8 months), 11.8% patients showed excellent response (ER), 17% SiR, while BiR/InR persisted in 71.2%. Less than half (38.5%) of SiR patients had radioiodine-avid metastases. Patients who, following the second ^131^I treatment, experienced SiR had larger tumor size and more frequently aggressive histology and vascular invasion than those experienced BiR/InR and ER. Also, the median values of thyroglobulin on levothyroxine therapy (LT4-Tg), Tg peak after recombinant human TSH stimulation (rhTSH-Tg) and thyroglobulin antibodies (TgAb) were significantly higher in patients who developed SiR. At last evaluation (median: 9.9 years), BiR/InR persisted in 57.5%, while 26.2% and 16.3% of the patients showed ER and SiR, respectively. About half of BiR/InR patients (71/153 (46.4%)) received further treatments after the second ^131^I treatment.

**Conclusions:**

Radioiodine-avid metastatic disease detected by the second ^131^I is an infrequent finding in patients with BiR/InR after initial treatment. However, specific pathologic and biochemical features allow to better identify those cases with higher probability of developing SiR, thus improving the clinical effectiveness of performing a second ^131^I treatment.

## Introduction

The main objective of the initial treatment of differentiated thyroid cancer (DTC) is to cure the patients and to reduce the risk of persistent/recurrent disease (1). However, in several patients, persistence or recurrence of the disease is detected during the follow-up. According to the suggestions of the most recent ATA 2015 guidelines (1), the risk of recurrence should be redefined at each clinical control according to clinical, biochemical, and imaging evaluations. If in patients with an excellent response (ER) to the initial treatment the risk of recurrence over time is almost negligible, in case of biochemical incomplete and indeterminate response (BiR/InR), it can vary according to several epidemiologic and pathologic factors, including the ATA class of risk. BiR/InR were defined according to ATA 2015 guidelines as follows: BiR in case of thyroglobulin value on levothyroxine therapy (LT4-Tg) ≥1 ng/mL or Tg peak after recombinant human TSH stimulation (rhTSH-Tg) ≥10 ng/mL or rising thyroglobulin antibodies (TgAb) levels with negative imaging; InR were defined by LT4-Tg <1 ng/mL or rhTSH-Tg <10 ng/mL or stable/declining TgAb levels with non-specific findings on imaging or faint radioiodine uptake in thyroid bed (1). The rate of BiR/InR after initial treatment is usually observed in 11% of low-risk, 2% of intermediate-risk, and 18% of high-risk patients, according to ATA risk stratification system (2). Patients who experience BiR after initial treatment have a risk of structural disease of about 20% during the follow-up; this rate decreases to about 15% in those who experienced InR (1). The management of patients with BiR/InR after the initial treatment is a matter of debate since they showed the persistence of Tg or TgAb, but without evidence of structural disease. Despite the suggestions of the ATA consensus guidelines (1), an attitude in managing these patients, common in clinical practice, is to perform an empiric treatment with radioiodine (^131^I) (3), although data about the efficacy of this treatment are controversial (4, 5, 6, 7, 8). It is worth noting that in nontertiary referral centers, with relatively low experience about the treatment of DTC, a delay in the application of the guidelines in clinical practice is reported (9). Moreover, the concept to set to zero the Tg/TgAb values is not completely abandoned.

The aim of this study was to verify the impact of performing a second ^131^I treatment in a cohort of DTC patients with BiR/InR after the initial therapy and unveil the factors able to identify the subgroup of patients who will develop structural disease for whom the treatment can be useful.

## Patients and methods

In the period between January 2009 and December 2012, 230 consecutive patients, after the initial treatment for DTC, were considered for a second ^131^I treatment at the Unit of Endocrinology of the University Hospital of Pisa. Ten out of 230 (4.3%) patients were excluded from the study because an additional neck surgery was performed in the time elapsed between the first and the second ^131^I treatment. Moreover, for the aim of the study that needs to evaluate only patients with BiR/InR, 67/220 (30.5%) cases who were retreated with a second ^131^I course due to the detection of SiR after the first ^131^I treatment, were also excluded.

**Figure 1:**
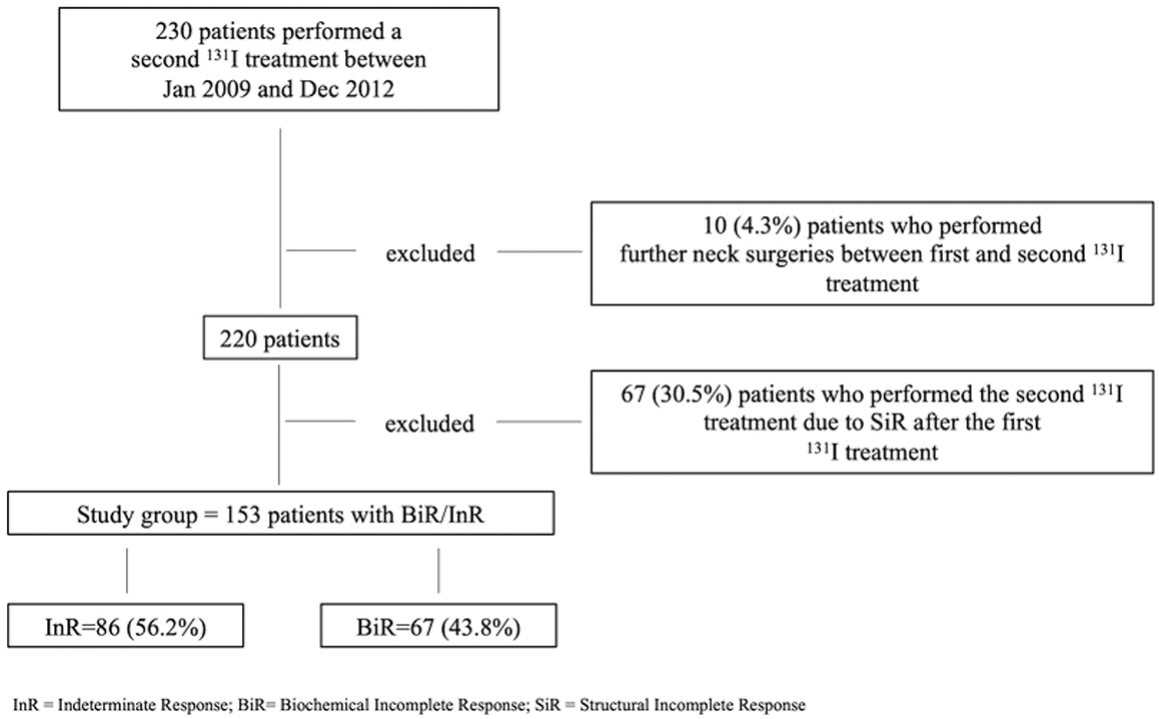
Study group selection criteria.

The remaining patients (*n* = 153) who experienced the second ^131^I treatment because of BiR/InR after the initial therapy represented the final study group (Fig. 1). The status of BiR/InR was defined after a median time of 7.5 (IQR: 6–10) months from the first ^131^I treatment. Of note, most of the patients already had a BiR (37.9%) and InR (56.9) at the first evaluation after initial treatment (median 7 (IQR: 6–9) months after the first ^131^I). Conversely, at this time 8 (5.2%) patients showed an ExR and became BiR/InR only later.

For each patient, demographic, pathologic, biochemical, and clinical data were collected. The TNM stage was reevaluated according to the eighth edition (10) while the class of risk applied at the time of the initial treatment was in accordance with the 2009 ATA Guidelines (11). The patients were followed up to the data lock of the study (August 2022). For the policy of the University Hospital, all patients signed a written informed consent to use their clinical and biochemical data for research purposes. This study was conducted in accordance with regulations of the Helsinki Declaration and was approved by the local ethical committee (CEAVNO – Comitato Etico Area Vasta Nord Ovest).

### Initial treatment

Total thyroidectomy was performed in all patients. Since, in our institution, we do not perform prophylactic lymph node dissection, central compartment (CCND) and/or laterocervical compartment (LCND) lymph node dissection were performed if lymph node metastases were identified before surgery at neck ultrasound (US) and confirmed through fine needle aspiration cytology (FNAC) and evaluation of Tg on washing fluid, or if suspicious lesions were found by surgeons during the surgery.

After surgery, all patients were considered for the treatment with ^131^I, using low (30 mCi – 1110 Mbq) or high (100 mCi – 3700 Mbq) activities, either with recombinant human thyroid-stimulating hormone (rhTSH) administration (i.m. injection of 0.9 mg of rhTSH for 2 consecutive days) or 30 days of LT4 withdrawal. The posttherapeutic whole-body scan (ptWBS) was performed after ^131^I in all cases through a one-head gamma camera (Aspex SPX 4000, Elscint Italy) with a high-energy collimator and a sensitivity of 160 cpm/mCi.

### Follow-up strategy

After the initial treatment, all patients were followed up over time according to the referral guidelines (1, 12). Blood evaluation for TSH, Tg, TgAb, as well as neck US, were routinely performed at each clinical evaluation. The imaging procedures, relevant for the right definition of BiR/InR patients, particularly total body CT scan and/or ^18^FDG-PET scan, were performed after the first evaluation in which positive Tg and/or TgAb values were detected. Further imaging procedures were performed during the follow-up only in cases of biochemical progression of the disease (both Tg and TgAb) according to the guidelines used at the time of the evaluation of the patients (1, 11).

The response to treatment, defined as ER, BiR/InR, and SiR, was applied to all patients (before and after 2015) according to the 2015 ATA Guidelines, for all the duration of the study and at each follow-up evaluation (1).

### Measurement of serum TSH, Tg, and TgAb

TSH measurement was performed by using an ultrasensitive immunochemiluminescent assay (Immulite 2000 TSH; Diagnostic Products Corporation, Los Angeles, CA, USA). The Tg measurement was performed with a second-generation immunochemiluminometric assay (Beckman Coulter, Fullerton, CA, USA; functional sensitivity 0.1 ng/mL) in all cases, but that performed during rhTSH stimulation that was evaluated by an immunometric assay (IMMULITE 2000 Thyroglobulin, Diagnostic Products Corporation, Los Angeles, CA, USA; functional sensitivity equal to 0.5 ng/mL determined in our laboratory).

TgAb were measured with immunofluorimetric method (AiaPack TgAb Tosoh Corporation, Tokyo, Japan) with a normal range between 0 and 30 U/mL and a Tg interference value of 8 UI/mL, determined in our laboratory (13).

### Neck US

Neck US was performed by endocrinologists with more than 5 years of experience in ultrasonography using a 7.5–12 MHz multifrequency linear probe. During the study period, several color Doppler equipment were used: from 2009 to 2012 AU 590 Asynchronous (Esaote Biomedica, Firenze, Italy), from 2013 to 2018 MyLab 50 (Esaote Biomedica, Florence, Italy) and since 2019 MyLab Twice (Esaote Biomedica, Florence, Italy). Ultrasonographic suspicious lymph nodes were evaluated by FNAC and Tg assay on washing fluid.

### Statistical analysis

Categorical variables are counted as a number and a percentage. Continuous variables are expressed as median and interquartile range. Categorical variables and continuous numerical variables are compared by chi-square test and Mann–Whitney *U*-test, respectively. A *P*-value less than 0.05 was considered statistically significant. Statistical analysis was performed by Statistical Package for the Social Sciences Statistics (version 20.0, Armonk, NY: IBM Corp).

## Results

In Table 1, demographic, pathologic, and clinical features of study group are shown. Most patients (67.3%) were females and the median age at diagnosis was 41 years. The median tumor size was 1.8 cm. The most frequent histology was classic variant of papillary thyroid carcinoma (CV-PTC) (60.8%). Tumor was multifocal in 90 cases (58.8%), and bilateral in about two-thirds of these (64.4%). Minimal extrathyroidal extension (mETE) was observed in 61.4% of cases while vascular invasion was less frequently detected (17.6%). According to the TNM eighth edition, most of cases were T1b (32.7%), and T2 (25.5%), while only seven patients (4.6%) showed a T4 stage. Lymph node dissection was not performed in half of the cases (Nx: 78 – 51%). In the remaining, CCND was performed in 32 (20.9%) cases, LCND in 7 (4.6%) cases, and both CCND and LCND in 36 (23.5%) cases. Among the patients who had lymph node dissection performed, 40 (53.3%) were classified as N1b, 27 (36%) as N1a, while the remaining 8 (10.7%) had no lymph node metastases (N0). According to the AJCC staging, most patients (86.9%) were in stage I, 11.1% in stage II, and none in stage IV. According to the 2009 ATA Guidelines, most of the patients (60.1%) were classified as intermediate risk, 36.6% as low risk, and only 3.3% as high risk of recurrence.

**Table 1:** Demographic, pathologic, and clinical features of the 153 patients who experienced the second ^131^I treatment due to BiR/InR.

Characteristics	Values,* n* (%)
Sex	
Male	50 (32.7)
Female	103 (67.3)
Age at diagnosis (years)	
<55	116 (75.8)
≥55	37 (24.2)
Median (IQR)	41 (29–53.5)
Tumor size (cm)	
≤1	37 (24.2)
1.1–2	58 (37.9)
2.1–4	42 (27.5)
>4	16 (10.5)
Median (IQR)	1.8 (1.1–2.6)
Histology	
CV-PTC	93 (60.8)
FV-PTC	30 (19.6)
FTC	3 (2)
AV-PTC	27 (17.6)
Multifocality	
Yes	90 (58.8)
No	63 (41.2)
Bilaterality	
Yes	58 (64.4)
No	32 (35.6)
mETE	
Yes	94 (61.4)
No	59 (38.6)
Vascular invasion^d^	
Yes	27 (17.6)
No	116 (75.8)
T stage^a^	
Tx	1 (0.7)
T1a	39 (25.5)
T1b	50 (32.7)
T2	39 (25.5)
T3	17 (11.1)
T4	7 (4.6)
Lymph node dissection	
Not performed	78 (51)
CCND	32 (20.9)
LCND	7 (4.6)
CCND+ LCND	36 (23.5)
N stage^a^	
Nx	78 (51)
N0	8 (5.2)
N1a	27 (17.6)
N1b	40 (26.1)
AJCC stage^b^	
I	133 (86.9)
II	17 (11.1)
III	3 (2)
IV	-
ATA class of risk^c^	
Low	56 (36.6)
Intermediate	92 (60.1)
High	5 (3.3)
First ^131^I treatment	
30 mCi	107 (69.9)
100 mCi	46 (30.1)
Median activity (IQR)	30 mCi (30–100)
L-T4 withdrawal	85 (55.6)
rhTSH	68 (44.4)

^a^According to TNM eighth edition; ^b^According to eighth edition; ^c^According to 2009 guidelines [11]); ^d^Data not available in ten (6.5%) cases.

AV-PTC, aggressive variant papillary thyroid carcinoma (tall cell variant, diffuse sclerosing variant, solid/trabecular variant, insular thyroid carcinoma); CCND, central compartment lymph node dissection; CV-PTC, classical variant papillary thyroid carcinoma; FTC, follicular thyroid carcinoma; FV-PTC, follicular variant papillary thyroid carcinoma; LCND, latero-cervical compartment lymph node dissection; mETE, minimal extrathyroidal extension.

At the time of the first treatment with radioiodine, about 70% of patients were treated with low dosage (30 mCi) of ^131^I. The appropriate TSH values for the treatment (>30 mUI/L) was obtained by LT4 withdrawal in 55.6% of patient and by rhTSH administration in the remaining 44.4%.

### Insights regarding the second ^131^I treatment in BiR/InR patients

The second ^131^I treatment was performed after a median time of 20 (IQR: 13.5–24) months and a median number of 2 (IQR: 1–2) clinical evaluations after the first one (Table 2). Most of patients (122 – 79.7%) received the second ^131^I treatment after LT4 withdrawal (Table 2).

**Table 2:** Insights regarding the second ^131^I treatment in the whole group (*n* = 153) of BiR/InR patients. Data are presented as *n* (%) or as median (IQR).

	Total	BiR	InR
*n*	153	67	86
Time between first and second ^131^I treatment, months	20 (13.5–24)		
Number of clinical evaluations between first and second ^131^I treatment	2 (1–2)		
Reason for performing the second ^131^I			
LT4-Tg	58 (37.9%)	48 (71.6%)	10 (11.6%)
Tg value (µg/L)	2.13 (1.3–3.7)	2.5 (1.6–4.9)	0.5 (0.37–0.95)
rhTSH-Tg	72 (47.1%)	11 (16.4%)	61 (71%)
Tg value (µg/L)	3.51 (2.55–8.6)	24.3 (19.7–46.1)	3.35 (2.28–7.23)
TgAb	23 (15%)	8 (12%)	15 (17.4%)
TgAb value (IU/mL)	265 (125–623)	550 (203–700)	164 (73–568)
Second ^131^I treatment	130 mCi (100–130)		
100–130 mCi	120 (78.4%)		
131–150 mCi	28 (18.3%)		
>150 mCi	5 (3.3%)		
LT4 withdrawal	122 (79.7%)		
rhTSH	31 (20.3%)		

Biochemical parameters (i.e. Tg and TgAb) of BiR/InR group performing the second ^131^I treatment were reported in Table 2. Most of the patients (78.4%) were treated with an activity ranged between 100 and 130 mCi.

### Clinical response after the second ^131^I treatment

The response to the second ^131^I treatment was defined at the first following evaluation (median time 8 months – IQR 7–10). According to the dynamic risk restratification system (2), this treatment led to an ER in 11.8% (*n* = 18), SiR in 17% (*n* = 26) while BiR/InR persisted in 71.2% (*n* = 109) of the cases.

**Figure 2:**
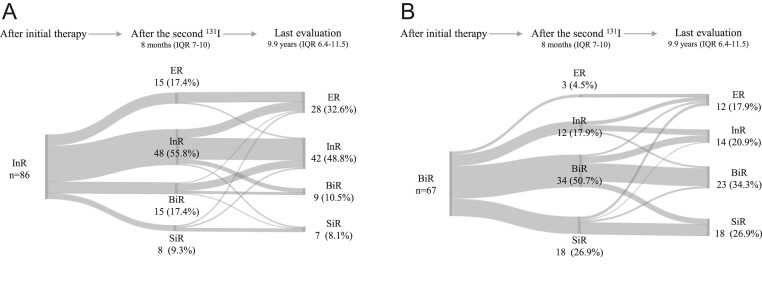
Changes of clinical responses over time in InR patients (*n* = 86; panel A) and BiR patients (*n* = 67; panel B).

It is worth to note that, according to the standard of care, some patients had further imaging procedures performed in the interval time between the second ^131^I treatment and the first evaluation thereafter. Therefore, not in all BiR/InR patients developing SiR, the detected metastases showed uptake of radioiodine. Indeed, in the group of 26 BiR/InR who showed SiR after the second ^131^I treatment, only ten patients (38.5%) showed radioiodine-avid metastatic disease (seven lymph nodes and three lung metastases). The remaining 16 (61.5%) patients did not show any ^131^I uptake in the metastases, that were discovered with other imaging methods. Overall, 6.5% (10/153) of the BiR/InR cases showed radioiodine-avid metastases after the second ^131^I treatment. Also, when dividing patients in InR and BiR, as expected ER was more frequently experienced in InR than BiR (17.4 vs 4.5%) as far as SiR in BiR than InR (26.9 vs 9.3%) (Fig. 2).

#### Pathologic features able to identify the subgroup of metastatic patients after the second ^131^I treatment

The comparison of the pathologic features in patients defined ER, BiR/InR and SiR after the second ^131^I is reported in Table 3. Patients with SiR showed a median tumor size (2.5 cm) significantly larger than BiR/InR (1.7 cm) and ER (1.4 cm) (*P* = 0.01). Regarding histologic variant, aggressive variants of PTC (AV-PTC) were more frequent in SiR (34.6%) compared with BiR/InR (14.7%) and ER (11.1%) (*P* < 0.01). Interestingly, vascular invasion was absent in patients with ER, while in BiR/InR and SiR group was described in 17.3% and 39.1%, respectively (*P* < 0.01).

**Table 3:** Comparison of demographic, pathologic, and clinical features in the 153 patients according to the clinical response (ER, BiR/InR, and SiR) after the second ^131^I treatment.

Characteristics	ER	BiR/InR	SiR	*P*
Total, *n* (%)	18 (11.8)	109 (71.2)	26 (17)	
Sex				0.37
Male	4 (22.2)	35 (32.1)	11 (42.3)	
Female	14 (77.8)	74 (67.9)	15 (57.7)	
Age at diagnosis (years)				0.93
<55	14 (77.8)	83 (76.1)	19 (73.1)	
≥55	4 (22.2)	26 (23.9)	7 (26.9)	
Median (IQR)	43.5 (31.75–54.75)	40 (29–52)	43 (26–59.75)	0.81
Tumor size (cm)				0.17
≤1	6 (33.3)	28 (25.7)	3 (11.5)	
1.1–2	8 (44.4)	41 (37.6)	9 (34.6)	
2.1–4	4 (22.2)	30 (27.5)	8 (30.8)	
>4	*–*	10 (9.2)	6 (23.1)	
Median (IQR)	1.4 (0.75–2.2)	1.7 (1–2.5)	2.5 (1.62–4)	0.01
Histology				<0.01
CV-PTC	8 (44.4)	73 (67)	12 (46.2)	
FV-PTC	7 (38.9)	20 (18.3)	3 (11.5)	
FTC	1 (5.6)	–	2 (7.7)	
AV-PTC	2 (11.1)	16 (14.7)	9 (34.6)	
Multifocality				0.34
Yes	13 (72.2)	64 (58.7)	13 (50)	
No	5 (27.8)	45 (41.3)	13 (50)	
Bilaterality				0.91
Yes	8 (61.5)	41 (64.1)	9 (69.2)	
No	5 (38.5)	23 (35.9)	4 (30.8)	
mETE				0.89
Yes	12 (66.7)	66 (60.6)	16 (61.5)	
No	6 (33.3)	43 (39.4)	10 (38.5)	
Vascular invasion^d^				<0.01
Yes	–	18 (17.3)	9 (39.1)	
No	16 (100)	86 (82.7)	14 (60.9)	
T stage^a^				0.17
Tx	–	–	1 (3.8)	
T1a	6 (33.3)	30 (27.5)	3 (11.5)	
T1b	7 (38.9)	36 (33)	7 (26.9)	
T2	4 (22.2)	28 (25.7)	7 (26.9)	
T3	1 (5.6)	11 (10.1)	5 (19.2)	
T4	–	4 (3.7)	3 (11.5)	
Lymph node dissection				0.35
Not performed	10 (55.6)	55 (50.5)	13 (50)	
CCND	3 (16.7)	20 (18.3)	9 (34.6)	
LCND	–	7 (6.4)	–	
CCND+ LCND	5 (27.8)	27 (24.8)	4 (15.4)	
N stage^a^				0.48
Nx	10 (55.6)	55 (50.5)	13 (50)	
N0	2 (11.1)	4 (3.7)	2 (7.7)	
N1a	2 (11.1)	18 (16.5)	7 (26.9)	
N1b	4 (22.2)	32 (29.4)	4 (15.4)	
AJCC stage^b^				0.83
I	16 (88.9)	96 (88.1)	21 (80.8)	
II	2 (11.1)	11 (10.1)	4 (15.4)	
III	–	2 (1.8)	1 (3.8)	
IV	–	–	–	
ATA class of risk^c^				0.14
Low	7 (38.9)	40 (36.7)	9 (34.6)	
Intermediate	11 (61.1)	67 (61.5)	14 (53.8)	
High	–	2 (1.8)	3 (11.5)	

^a^According to TNM eighth edition; ^b^According to eighth edition; ^c^According to 2009 guidelines [11]); ^d^Data not available in ten (6.5%) cases.

AV-PTC, aggressive variant papillary thyroid carcinoma (tall cell variant, diffuse sclerosing variant, solid/trabecular variant, insular thyroid carcinoma); CCND, central compartment lymph node dissection; CV-PTC, classical variant papillary thyroid carcinoma; FTC, follicular thyroid carcinoma; FV-PTC, follicular variant papillary thyroid carcinoma; LCND, latero-cervical compartment lymph node dissection; mETE, minimal extrathyroidal extension.

#### Biochemical features able to identify the subgroup of metastatic patients after the second 131I treatment

Among the 58 patients receiving the second ^131^I treatment for detectable LT4-Tg, the median values were significantly higher in patients who developed SiR (3.73 µg/L) than in those with persistent BiR/InR (2.05 µg/L) (*P* = 0.04). Only three patients among those receiving the second ^131^I treatment for detectable LT4-Tg had ER.

Among those patients who received the second ^131^I treatment due to the stimulated Tg value after rhTSH (*n* = 72), a significant increasing trend of the median Tg peak value (*P* = 0.03) was observed passing by patients experienced an ER (3.31 µg/L) to Bir/InR (3.75 µg/L) up to SiR (8.85 µg/L).

In the patients re-treated for persistence of TgAb (*n* = 23), none showed ER after the second ^131^I treatment. However, the median value of TgAb was significantly higher (*P* = 0.04) in patients experienced SiR (1295.5 UI/mL), than in those with BiR/InR (210.2 UI/mL) (Table 3).

### Clinical response at last evaluation

The study cohort was followed for a median time of 9.9 (IQR: 6.4–11.5) years from the initial diagnosis and 7.6 (IQR: 4.6–9.7) years from the time of the second ^131^I treatment.

**Figure 3:**
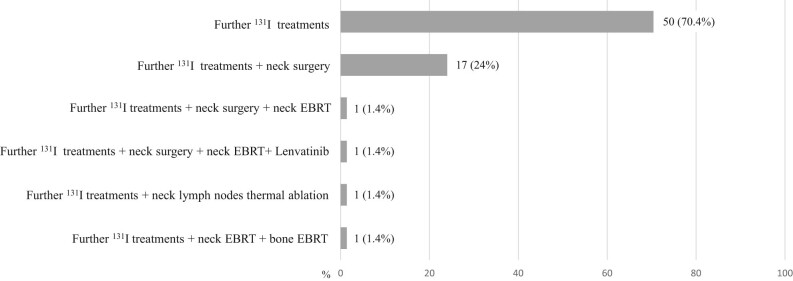
Types of additional treatments performed after the second ^131^I (71/153 patients (46.4%)).

Eighty-two patients (53.4%) performed only a second ^131^I after the initial treatment, without receiving any other treatment during the follow-up. The remaining (71/153 (46.4%)) received other ^131^I and/or other types of treatment as reported in Fig. 3.

**Figure 4:**
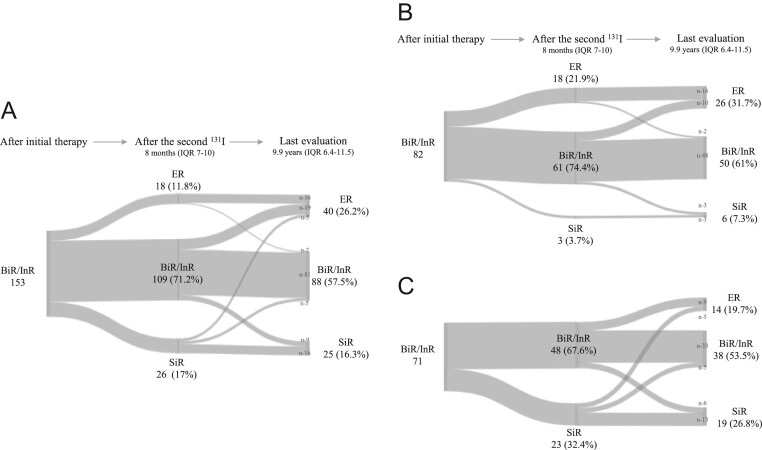
Changes of clinical responses over time in all patients of the study group (*n* = 153; panel A), and divided in those not receiving (*n* = 82; panel B) and receiving (*n* = 71; panel C) further treatments after the second ^131^I treatment.

Fig. 4, panel A, shows how clinical responses changed over time in the whole group of patients. At last evaluation the total number of ER was 40/153 (26.2%) demonstrating a relevant increase compared to the ER prevalence after the second ^131^I treatment (18/153 (11.8%)). Almost half of patients who showed an ER at last evaluation (19/40 (47%)), belonged to the BiR/InR group after the second ^131^I treatment. Among them, 10 patients received other ^131^I treatments, while nine patients became ER without other active treatments during the follow-up. Moreover, five patients with ER at the end of the follow-up had SiR after the second ^131^I treatment; all of them experienced further treatments (i.e. ^131^I and neck surgeries) to achieve the ER. In more than half of patients, BiR/InR (88/153 (57.5%)) persisted at last evaluation: almost all of them (81/88) already had BiR/InR response after the second ^131^I treatment, while the other seven derived from either ER (*n* = 2) and SiR (*n* = 5). Twenty-five patients (16.3%) showed an SiR at last evaluation: 16 of them were already SiR after the second ^131^I treatment, while nine patients belonged to the cohort of BiR/InR patients. The trend of how the clinical outcome was modified over time in patients not submitted or submitted to further treatments, is shown in Fig. 4B and C, respectively. Similar results to those reported at the first evaluation after the second ^131^I treatment were also shown at last evaluation when splitting the whole group in InR and BiR (Fig. 2). Overall, during the study period 35/153 (22.9%) patients experienced SiR and the median time of SiR detection was 8 (IQR: 6–21.7) months after the second ^131^I treatment.

## Discussion

The use of ^131^I in patients with DTC has been widely revised in last years (1). Differently from the past, ^131^I was less and less frequently used for the treatment of DTC both after surgery and consequentially during the follow-up. Conversely, additional ^131^I treatments are still strongly recommended in patients with radioiodine-avid metastatic disease for therapeutic purposes (1).

In the recent past, and in many centers further ^131^I treatments after the first one have been performed also in case of BiR/InR patients, with the aim to set to zero the values of Tg and TgAb or to treat potential metastatic lesions not detected before (4, 5). However, scanty data were reported about the real benefit of further ^131^I in patients with BiR/InR, particularly, about the outcome of the second ^131^I performed after the initial treatment.

In this study, we reported the data about the short and long-term clinical response of patients with DTC who showed a BiR/InR after the initial treatment and were re-treated with a second ^131^I course.

After a median time of 8 months from the second treatment with ^131^I, about 12% of these patients became either serum Tg or TgAb negative and were restaged to ER, suggesting a clinical benefit of this treatment. This rate increased up to 26.1% in a long-term follow-up (median 9.9 years), interestingly also in cases without further treatments.

Several studies reported a spontaneous decline of Tg and TgAb values over time, in the absence of further treatments, even in patients in whom radioiodine treatment was never performed (2, 13, 14, 15). However, at variance with the aforementioned studies, in our series only ten out of 61 (16.4%) patients who were already BiR/InR after the second ^131^I treatment reached the ER in a long-term follow-up (10 years), without any further treatment over time.

Moreover, it is relevant to highlight that, in the context of low and intermediate risk patients, the role of benign residual tissue in producing Tg or determining the persistence of TgAb, cannot be excluded. This is particularly relevant in patients treated by not expert surgeons, who are not submitted to ^131^I treatment (16), depending by the TSH stimulation on the residual tissue more than a real persistent/recurrent disease (17). Because of the high sensitivity of the Tg assays, is not unusual that low levels of Tg are present also in patients treated with radioiodine. In all these cases, the trend of serum Tg or TgAb values is more relevant than absolute value *per se*.

Compared to ours, several studies reported higher rates of ER in a medium long-term follow-up in patients with initial BiR/InR, not receiving any additional treatment (18, 19, 20). Our lower ER rate, both in the short- and long- term follow-up, might be due to the inclusion of patients with higher values of Tg and/or positive TgAb, not included in other series.

Our data showed that BiR/InR persisted as the most common response both after the second ^131^I treatment and at last evaluation, with a similar rate between patients receiving or not additional treatments. Similar results were showed by Hirsch *et al.* (21) who demonstrated that most of their patients continued to have detectable Tg values after 1–2 years from the second ^131^I treatment, and at last evaluation performed about 10 years after initial treatment. However, it is worth noting that they included patients selected across 20 years and differently treated (i.e. higher activities of ^131^I as first treatment) over time and this could be responsible of some of unvoluntary bias.

After the second ^131^I treatment, 26/153 (17%) of our patients showed structural disease, but it is worth to note that only ten of them, corresponding to 6.5% of the total series, showed metastatic lesions detected at the ptWBS, while the others were found with other imaging techniques. Likewise, Hirsch *et al.* reported 15.5% of structural disease within 2 years after the second ^131^I course in patients with BiR after initial treatment. However, in this study the metastatic lesions, described mainly in the neck, were not confirmed with cytology and no information about their ability to take up radioiodine were provided. Conversely, all metastatic lesions described in our study have been confirmed by either FNAC or uptake of radioiodine at ptWBS.

The rates of radioiodine-avid disease detected after the second ^131^I treatment in settings like ours are rather variable. Kim *et al.* (7) did not find ^131^I uptake in any of the 14 patients receiving the second ^131^I treatment for Tg values ≥10 µg/L and negative imaging. A low rate of metastatic disease was observed by Rosario *et al.* (22) who detected radioiodine-avid lung metastases in only one out of 24 patients receiving an empiric second ^131^I treatment due to detectable Tg values but with negative imaging. Conversely, other studies showed a higher rate of radioiodine-avid lesions detected after the second ^131^I treatment (25–94%) (5, 6, 23). The results of these studies are not fully comparable to ours since performed several years ago, when the diagnostic WBS was commonly performed before retreating patients with therapeutic activities of ^131^I. Thus, a not negligible number of cases with detectable Tg and negative diagnostic WBS, were indeed affected by structural disease already present at the time of the first ptWBS. In our study, these patients have been excluded and only cases with negative imaging after the first ^131^I treatment have been included.

As previously said, in 16 cases the detection of structural disease did not arise by the treatment with ^131^I but was discovered during the time elapsed between the second ^131^I treatment and the subsequent clinical evaluation, by other imaging modalities (i.e. US, CT scan, MRI). Particularly, in 15/16 cases, lymph nodes metastases were detected by neck US, confirmed by cytology and evaluation of Tg on washing fluid, without showing uptake after the second ^131^I treatment. The presence of lymph nodes metastasis which do not show radioiodine uptake is not a rare finding. Indeed, Kim *et al.* (7) detected lymph node metastases by neck US in 5 out of 14 patients which did not show any uptake of ^131^I. Also, Rosario *et al.* (22), among the 24 patients receiving empirical ^131^I treatment, identified one patient with lymph node metastases by neck US and another with bone metastases diagnosed by CT and ^18^FDG-PET scan, both without uptake of ^131^I. These findings demonstrated that most of the metastatic lesions in patients with BiR/InR after initial treatment are unable to take up radioiodine leading to an ineffective second ^131^I course. However, it is somehow useful to define the radioiodine refractory status of these lesions, a key point in considering other treatments if needed (24). ^18^FDG-PET scanning found a role to localize structural disease in Tg-positive and WBS-negative patients (25, 26), particularly in high risk DTC patients with serum Tg levels >10 ng/mL (1). However, in our cohort, consisting of more than half of InR patients, only few cases had indication to perform ^18^FDG-PET scanning.

Several papers showed a good performance of ATA risk stratification system, based on histology, in predicting the potential persistence/recurrence of the disease. Also in our series, we observed a positive association between SiR occurrence after the second ^131^I treatment and larger tumor size, aggressive histology, and vascular invasion. These features were already known to be negative prognostic factors for recurrence in DTC patients in several series (27, 28, 29, 30, 31, 32); thus, they must be taken into consideration even in the decision making to repeat or not the second ^131^I course in patients with BiR/InR after the initial treatment.

In patients without interfering TgAb, Tg values can be considered as a prognostic factor (1). Indeed, although Tg value is influenced by several factors (i.e. residual thyroid cancer and/or normal thyroid tissue, TSH level, functional sensitivity of the assay, iodine status, etc.) other studies confirmed its predictive role for persistence/recurrence of disease (33, 34, 35, 36, 37, 38, 39). Less is known about the specific prognostic role of Tg value, evaluated before the second ^131^I course, in predicting the clinical response of these patients to the treatment. Our results showed that higher level of LT4-Tg and rhTSH-Tg before the second ^131^I treatment are associated with the persistence of BiR/InR or detection of SiR after the treatment. Moreover, patients who developed SiR showed higher values of both LT4-Tg and rhTSH-Tg compared with those who remained BiR/InR, after the second ^131^I treatment. Similar results have been reported by Hirsch *et al.* (21), although the patients in their series were followed for a shorter-term follow-up than ours and no correlation between serum Tg values and the response to treatment was performed. So far, there are no studies that specify any Tg cutoff value able to predict the response to the treatment; we also tried to find a potential LT4-Tg and rhTSH-Tg cutoff (data not shown), but the low sensitivity and specificity made our result ineffective. In this regard, we agree with Rosario *et al.* (22), who suggested that more than a single serum Tg value, an empiric ^131^I treatment should be restricted to patients showing a trend of increasing serum Tg values over time. The same concept can also be applied to patients with increasing titers of TgAb (40).

A non-negligible part of our study group (15%) consisted of patients with positive TgAb, therefore interfering with Tg values. In the short-term follow-up after the second ^131^I treatment, TgAb continued to be detectable in all patients, thus none of them could be considered in clinical remission and their condition of BiR/InR remained unchanged. Moreover, 5 (19.2%) patients with positive TgAb experienced a SiR after the second ^131^I treatment. Like that observed for Tg, these latter patients showed significantly higher TgAb values when compared with patients with BiR/InR.

Our study showed several limitations mainly due to its retrospective nature. First, the lack of a control group with BiR/InR in which the second ^131^I treatment was not performed, because almost all patients in the years in which the study group was selected performed empiric ^131^I treatments. Moreover, not all patients were treated with the same activity of radioiodine, already from the first treatment. However, the uniform modality of follow-up over time, with a clinical, biochemical and imaging evaluation performed in the same center for all the duration of the study, is a relevant strength.

## Conclusions

Our study indicates that the second ^131^I treatment in patients who had a persistent biochemical incomplete or indeterminate response after the surgery and first ^131^I treatment, should neither totally *a priori* avoided nor unconditionally performed in all cases. The presence of detectable Tg or TgAb values alone is not necessarily indicative of radioiodine-avid structural disease, as our data demonstrated that only a minority of cases who underwent the second ^131^I treatment showed radioiodine uptake in the metastatic lesions. Therefore, better identification of those cases with more probability of having radioiodine-avid structural disease after the second ^131^I treatment, should consider the poor prognostic factors at diagnosis like tumor size, aggressive histology, and vascular invasion, as well as the higher values of Tg and/or TgAb. All the other cases should be carefully monitored with the evaluation of the Tg and TgAb trend and imaging techniques over time, mainly neck US and CT scan with i.v. contrast, and only if structural disease is detected, a further ^131^I treatment should be performed to demonstrate (or not) the radioiodine refractory status.

## Declaration of interest

R Elisei is a consultant for EISAI, Lilly, Ipsen, and Bayer and Antonio Matrone is a consultant for Lilly, but this study was not influenced by this activity. The other authors declare that there is no conflict of interest that could be perceived as prejudicing the impartiality of the research reported.

## Funding

This study has been supported by grants to RE from Associazione Italiana per la Ricerca sul Cancro http://dx.doi.org/10.13039/501100005010 (AIRC, Investigator grant 2018, project code 21790).

## Data availability

Some or all datasets generated during and/or analyzed during the current study are not publicly available but are available from the corresponding author on reasonable request.
